# The African continent should consider a harmonized consultative and collaborative effort towards coordinated policy and regulatory guidelines across the fields of biotechnology

**DOI:** 10.3389/fbioe.2023.1211789

**Published:** 2023-06-07

**Authors:** Tlou Samuel Masehela, Eugenia Barros

**Affiliations:** ^1^ South African National Parks, Scientific Services, Skukuza, South Africa; ^2^ EB Biosciences and Consulting, Sandton, South Africa

**Keywords:** Africa, biotechnology, biosafety, regulatory guidelines, policy, convention on biological diversity (CBD), genome editing, new breeding technologies (NBTs)

## Abstract

The advances in the field of biotechnology (and bioengineering) over the past decades has allowed the precise development of new products across the agricultural, environmental, and pharmaceutical sectors. This has led to the need to evaluate the relevance and applicability of existing policies and frameworks that regulate the current transgenic technologies. On the African continent, there are delays in the development and implementation of biosafety policies and regulations. Most African countries formulate their policies, regulations, and frameworks by following The Convention on Biological Diversity’s (CBD) guidelines. Although the CBD documents are continually evolving, this happens at a slower pace. It is becoming increasingly important for countries to deal swiftly with the advances in biotechnology in a manner that *balances* the regulatory complexities, while safeguarding the *net gains* for human health, the environment, and the economy. For the African countries, some of these *net gains* are similar, while concerns and perceived risks associated with the adoption and use of the technology are also common. Furthermore, the challenges relating to capacity, knowledge, and skills to address some of the regulatory complexities. In this article we explore the advancement of some African countries in the development and implementation of various biosafety policies and detail the challenges and constraints faced by those countries that are lagging behind. We conclude by outlining identified opportunities for neighbouring and regional countries to assist one another and work in a more organised and coordinated approach towards developing, implementing, and strengthening their respective biosafety policies, regulations, and frameworks.

## Introduction

The field of biotechnology has overtime been recognised to be rapid in terms of new improvements and advancements towards supporting innovation across the different fields of research and development (Barragán-Ocaña, 2020; Ma, 2021). The significant potential for their applications cuts across many fields and disciplines, with the major ones being agriculture and health (medicine). In these two fields, biotechnology has presented to the human population several useful products by using enzymes, microbes, proteins, and various metabolic machinery of plants and animals (Masson et al., 2001; Khan, 2014; Pham, 2018).

The biggest impact of biotechnology has been in the field of agriculture mainly because of the need for more sustainable food production to feed the ever-increasing world population (Giller et al., 2021). Working with agricultural farmers, scientists have developed biotechnology tools to complement conventional crop improvement methodologies to produce genetically modified crops (GMOs). These crops are better adapted to grow in different environments, to be more resistant to agricultural biotic and abiotic stresses, to be better protected against pests and to have improved nutritional quality (Tran et al., 2010; Abdallah et al., 2014; Kamthan et al., 2016). The latest plant-breeding technology tool that has the potential to revolutionize agriculture is the development of genome edited crops. If the African continent is to benefit from these biotechnology developments there is an urgent need for discussion, debate, and harmonization of guidelines across the continent.

The adoption, application, and use, of biotechnology has not always been positive, as it has been marked with various concerns and controversies (Bauer, 2002). The debates on this subject comes mainly from the public and goes as far back as the early introductions of Genetically Modified (GM) products (Hielscher et al., 2016). In their early years, Genetically Modified crops, and foods, were to a large degree met with different perceptions and a strong level of mistrust–especially those based on personal or religious beliefs (Phillips, 2008). In most instances, the discussions and perceptions remain highly emotional, and focused on the potential economic, environmental, human health and social risks (Carr & Levidow, 2000; Goyal & Gurtoo, 2011; Lucht, 2015). Although, the trend on concerns varies across the continents, common issues are centred around ethical standards of practice, the morality and unpredictable results that come with different gene manipulations and experiments (Deane-Drummond et al., 2001). In some instances, questions are raised around the impacts on small-scale farmers and communities when it comes to seed rights and the socio-economic implications (direct/indirect), issuing of patents, and the equitable sharing of some of the proceeds from the biological resources and genetic material derived from regions/countries (Masehela et al., 2021). Furthermore, arguments remain that the GM technology depicts and promotes a particular narrative around a solution towards the global food crisis focusing on crops and traits (Stone & Glover, 2011; Stone & Glover, 2017). At the same time, others argue that a lot of the debates and criticism of the technology discredits various benefits already achieved with its application and use (Klümper & Qaim, 2014; Smyth, 2020).

The dawn of GMOs on the African continent has forever been marked by the hesitancy to accept, emanating from unfavourable policies and a wide array of public opinions (Gbadegesin et al., 2022). Besides the general lack of knowledge base, education and awareness of the technology and its application to the public (Gastrow et al., 2018), undecisive political attitude to GMOs has also been noted to have added more confusion and indirectly increased mistrust within the technology space (de Cheveigné et al., 2002). It is for this reason that there has been calls for care-based approach to ethics and politics so that social, economic, and ethical considerations are strategically incorporated into biotechnology governance and regulatory assessments (Wickson et al., 2017). For the African continent, this is important given that public trust is critical for the technology’s success and its benefits to be realised. However, this does not mean that the longstanding concerns, implications, and questions around safety should be forgotten (Trump et al., 2022). We know now that the world has begun embracing New Breeding Technologies (NBTs), spearheaded by the likes of CRISPR-Cas9 and other gene editing techniques (de Graeff et al., 2019). Already, we are seeing several concerns and oppositions to these technologies across the world (Helliwell et al., 2017), and since the African continent has not fully advanced from its GMOs challenges and drawbacks, it might be difficult to advance to the new politics and governance of these new technologies.

Countries and governments across the globe have set up regulatory agencies (bodies and committees) that will have oversight and make decisions regarding the validity of the research, development and the safety in the application of the technology and its derived products (McLean et al., 2012; Komen et al., 2020; Turnbull et al., 2021). However, the level in which the various regulations, biosafety frameworks and policy instruments are designed, implemented, enforced, and monitored differs depending on the country/government needs (Cantley, 2007). The focus areas are to a large extend guided, shaped and controlled, by country priorities, political influences and leadership, and the economic elements. For those countries that are signatories and party to the Cartegena Protocol on Biosafety to the Convention on Biological Diversity (CBD), the treaty was and remains instrumental in providing guidance and governance on the movements of living modified organisms (LMOs) resulting from modern biotechnology (Glass, 2000). Subsequent, several supplementary protocols and agreements have been put in place, recognising that with the rapid advances in the field of biotechnology; there is a need to protect biological diversity from the potential risks posed by living modified organisms (Shibata, 2014). At the same time, these key protocols have had their own shortcomings as they have not fully kept up with the fast developments within the biotechnology space and this is evident with the lack of clear definitions and guidance in fields such as Synthetic Biology (Hokanson, 2019; Groenewald, 2021). Although this can be viewed as a drawback, it should not undermine the substantial work done over the years through the various committees, expert and working groups [e.g., Ad Hoc Technical Expert Group (AHTEG)] and online forums of the CBD.

One of the major challenges for countries/parties has been that of taking on the guidance documents, training manuals and other supplementary materials for further development in line with their country needs (Pertry et al., 2014). Often, this failure is attributed to the lack of political will, lack of financial resources, relevant expertise, knowledge and experience in the respective policy and framework areas (Kameri-Mbote, 2002; Falkner & Gupta, 2004). This is particularly true for the African continent and remains a great challenge for most countries–in turn, lack of progression when it comes to exploring the potential applications of biotechnology and its associated bioengineering tools (Makinde et al., 2009). In this article, we explore: 1) the relevance and applicability of agricultural biotechnology to the African continent; 2) review and outline African countries that have made good strides in developing relevant biosafety protocols towards regulating the use of the technology; 3) explore some of the drawbacks of progress or reluctance in formulating and implementing biosafety protocols; and 4) propose or put forward an approach that could benefit the continent towards achieving various components of their frameworks, policy and biosafety protocols for guidance when considering the adoption and use of biotechnology–and bioengineering tools/options.

## The context and relevance of biotechnology for the African continent

Biotechnology has a strong significance for the African continent in terms of contribution towards solving and/or offering options in mitigating a multitude of problems in both the agriculture and health sectors. Several studies do recognise the massive potential that biotechnology has to offer to the continent when it comes to improving agricultural production (Juma, 2015), improving economic growth, contributing to food and nutrition security (Binswanger-Mkhize et al., 2010; Kedir & Kararach, 2019), strengthening scientific capacity and advancement, providing alternative solutions to waste management, and improving health as well pharmaceutical options in the medicinal field (Bediako, 2022). The 2009 publication by the New Partnerships for Africa’s Development (NEPAD), outlines challenges facing the African continent on biotechnology and biosafety (Makinde et al., 2009). Among others, the report highlighted the financial challenges, the lack/loss of trained technical expertise; slow development of the biotechnology sector; inadequate Intellectual Property Rights infrastructure; lack of political will and government leadership. Today, these shortcomings remain prevalent and are evident in the lack of progress in biotechnology policy advancement and/or development of national biosafety frameworks (NBFs) across the continent.

Without these laws, regulations, guidelines, or policies related to biotechnology, it remains difficult to carry out or conduct any biotechnology related activities in the respective countries. Paarlberg (2009) indicates that one of the major constrains exploring new technologies in agriculture for Africa, stems from the lack of formulation–subsequently, implementation of relevant policies and regulations that would be geared towards agricultural advancement through science. In fact, they specifically cite the disapprovals on modern agricultural biotechnology because of inadequate policy frameworks to support its update. Similarly, Egwang (2001) and Bediako (2022), demonstrates that biotechnology has the potential to transform the health and the economies of most African countries, and that for this to be realised, African governments must create enabling environments through positive policies and the availability of resources.

African countries continue to face challenges when it comes to food production and medicinal needs (Pinstrup-Andersen & Watson, 2011). Countries find it difficult to provided adequate healthcare (and products/medicines), while farmers find it difficult to control and manage agricultural pests. At the same time, multilevel approaches are needed to overcome these challenges that are further exacerbated by increasing environmental, economic, and social challenges. Moreover, biotechnology has moved far beyond the *basic principles* of GMOs, offering some of the most powerful technological tools as options for mitigating most challenges and constraints in both agriculture and medical fields. Wambugu (1999), Machuka (2001), Nitin et al. (2022), Mfutso-Bengo & Muula (2007) and Sammut (2021) outline some of the potential benefits that can be realised for the African continent in agriculture and medicine, respectively.

## Brief overview of biosafety policies, regulations and/or frameworks for African countries

The regulatory landscape of genetically modified products in Africa is still very diverse and harmonization of its regulatory processes has not yet been archived. There are many obstacles facing the commercial release of GM crops and they include biosafety factors, public and farmer acceptance as well as, political will and support (Akinbo et al., 2021). The 55 member states of the African union have developed specific regulatory agencies to approve seed regulation and variety regulation of crops produced by conventional methodologies under the Seed Act in addition to a National Biosafety Authority (NBA) that regulates crops developed using biotechnological approaches, like GMOs. Under the Seed Act regulation many African countries require approval by the National Performance Trial Committee (NPTC) and the National Variety Release Committee (NVRC) for the release and commercialization of conventionally derived seeds. Regarding the environmental release and commercialization of GMOs, African countries are at different levels of adoption of GM crops and only a few have approved the commercial release of crops for farmer adoption. To consider a joint and co-ordinated regulatory guideline for the continent one needs to understand where they are at, what regulations are in place and where the regulatory process could be fast tracked. An outline of the process used by Kenya, Nigeria, Eswatini, Ethiopia, Ghana, Malawi, Mozambique, Sudan and South Africa is summarized in Table 1 (Akinbo et al., 2021). These countries have commercialized GM crops (e.g., Bt cotton) but have their own specific Seed Laws and Regulations, and follow different steps some of which maybe more laborious resulting in a fast or slow approval of GM crops.

**TABLE 1:** Regulatory processes adopted by different African countries (adopted and modified from Akinbo et al., 2021).

	Biosafety regulatory framework	Seed acts and implementing regulations
Kenya		
Laws and Regulations	Biosafety Act 2009 and implementing regulations to cover contained use, environmental release, import, export, and transit	Seed and Plant Varieties Act (Seed Act; Cap.326 (Gok, 2012) and the Seeds and Plant Varieties Regulations (NPT Regulations)
Agencies/Department	National biosafety Authority is the Competent Authority	KEPHIS, Ministry of Agriculture
Committees	Scientific Advisory Committee	National Performance Trial Committee National Variety Release Committee
Nigeria		
Laws and Regulations	National Biosafety Management Agency Act 2015 revised in 2019 to National Biosafety Management Agency Act 2019	National Agricultural Seeds Act, N5 Laws of Nigeria, 2004 revised to give National Seed Act (NSC) Act 2019
Agencies/Department	National biosafety Management Agency (NBMA) is the National Biosafety Authority	National Agricultural Seeds Council (NASC), an agency of the Federal Ministry of Agriculture and Rural Development
Committees/PARTNERSHIPS	The Nigeria Agricultural Seed Council; National Agricultural Quarantine Service; Nigeria Customs Service; National Agency for Food and Drug Administration and Control; Federal Ministry of Agriculture (Department of Veterinary and Pest Control); Standard Organization of Nigeria; Federal Competition and Consumer Protection Commission	National Crop Varieties and Livestock Breeds Registration and Release Committee
Eswatini		
Laws and Regulations	Biosafety Act of 2012 (under review)	Plant Control Act, 1981 (under review); Seeds and Plant Varieties Act of 2000 and Plant Varieties Regulations
Agencies/Department	Eswatini Environmental Authority	Seed Quality Control Services, under the Ministry of Agriculture
Committees	National Biosafety Advisory Committee	National Variety Release Committee
Ethiopia		
Laws and Regulations	Biosafety Proclamations (Proclamation No. 655/2009 and the Amendment into Proclamation No. 896/2015	Seed Proclamation (Proclamation No. 782/2013) revised to give Proclamation No. 206/2000 in 2000
Agencies/Department	Environment, Forest, and Climate Change Commission	National Seed Quality Control and Certification Division under MoARD
Committees	National Biosafety Advisory Committee	National Crop Improvement Committee
Ghana		
Laws and Regulations	Biosafety Act 831, 2011 and Implementing Regulations	Plants and Fertilizer act of 2010 (803)
Agencies/Department	National Biosafety Authority	National Crop Improvement Committee
Committees	Board consisting of experts in biotechnology and related biological sciences, including biosafety	Plant Protection and Regulatory Services Directorate
Malawi		
Laws and Regulations	Biosafety Act was passed in 2002 and implemented in 2007 and National Biotechnology and Biosafety Policy was enacted in 2008	Seed Act of 2005 and recently published seed Regulations 2018
Agencies/Department	National Biosafety Regulatory Committee (NBRC) is the Competent Authority	The Seed Services Unit of DARS (Department of Agricultural Research Services)
Committees	National Biosafety Regulatory Committee, which includes Reviewers, Inspectors and Biosafety Registrar	Agricultural Technology Clearing Committee (ATCC)
Mozambique		
Laws and Regulations	Decree no. 6/2007 (regulation) with an amendment in 2014 to allow for the commercialization of GMOs to give Decree 71/2014 of 28 November 2014	12/2013 Seed Regulation Decree
Agencies/Department	Minister of Science and Technology, Higher and Technical Vocational Education, is competent authority on matters pertaining to GMO approvals	National Seed Committee (NaSC) in Ministry of Agriculture and the Variety Registration and Release Committee
Committees	The Grupo Inter-Institucional Sobre Bio-Segurança, (GIBS) serve as advisory committee to the Minister of Science and Technology, Higher and Technical Vocational Education	Department of Seeds in the Ministry of Agriculture
Sudan		
Laws and Regulations	Biological Safety Act 2020	New Seed Law in 2009
Agencies/Department	Sudan National Biosafety Council (SNBC)	National Seed Council
Committees	—	—
South Africa		
Laws and Regulations	Genetically Modified Organisms Act 1977 (Act No.15 of 1997) revised in 2006 to Genetically Modified Organisms Act No. 23 of 2006	Plant Breeder’s Rights Act 1976 (Act No. 15 of 1976)
Agencies/Department	Formerly Minister for Agriculture, Forestry and Fisheries and now Minister of Agriculture, Land Reform and Rural Development	Formerly Minister for Agriculture, Forestry and Fisheries and now Minister of Agriculture, Land Reform and Rural Development
Committees	Advisory Committee (AC) and Executive Council (EC)	—

With the rapid advances in biotechnology, it is crucial for African countries to work together and try to harmonize their science-based regulatory guidelines to be ready for the release and approval of products developed using CRISPR/Cas9-mediated genome editing. CRISPR-Cas9-based genome editing has become the most prevalent genetic engineering approach to develop improved crop varieties in addition to conventional technologies due to its simplicity, precision, and accuracy (Arora & Narula, 2017; Montecillo et al., 2020). Genome editing technologies enable the targeted manipulation of plant genomes and therefore it speeds up the breeding processes enabling breeders to address urgent goals with greater precision (Ceasar et al., 2016; Rao & Wang, 2021). Although globally there is not yet a definite consensus on how to regulate genome editing products, some countries have opted to regulate genome-edited crops based on the presence/absence of foreign DNA integration. So, genome-edited crops that do not have any foreign gene and the edited gene is not harmful to other plants and its safety attributes are comparable to its conventionally bred crops, does not require regulatory evaluation. Likewise, genome-edited foods whose safety attributes are comparable to those produced by conventionally bred crops, do not require regulatory evaluation.

Here, we are not suggesting or advocating that the African continent take a limited oversight on gene edited products, but rather explore paths towards homogeneity within the regulatory space of these new technology-based products, in line with their country specific needs and economical advancements. We also note that the scope of the technology and its applications will continue to advance, and the flexibility to accommodate these future developments will be of great importance. Therefore, bringing into the spotlight the need for effective risk management, responsible governance, and a robust approach to regulatory coherence.

To date, Nigeria was the first African country to develop biosafety guidelines through the National Biosafety Management Agency (NBMA 2020) to regulate genome editing products followed by Kenya. Both countries have adopted a case-by-case biosafety regulations for genome-edited products. As a result, when the genetic manipulation process requires the use of recombinant DNA sequences or the genome-edited product has a novel combination of genetic material, the product will be regulated as a GMO. But if the genetic changes do not include foreign DNA and thus introduces genetic changes that are comparable to conventional breeding outcomes, the product will be treated as a non-GMO and are therefore exempt from GMO regulations. South Africa has adopted the approach that gene-edited products should be treated as GMOs and as such to be regulated as GMOs (DALRRD Public Notice, 2021). Since the CRISPR/Cas9 technology was discovered, many African countries have been using it in the improvement of the major staple food crops (Tripathi et al., 2022). Currently, Burkina Faso, Egypt, Ethiopia, Ghana, Kenya, South Africa, and Uganda are the only African countries with active projects that involve the use of gene editing techniques (Gakpo, 2021; Karembu, 2021; Sprink et al., 2022).

## Current efforts on policies and biosafety regulations development on the African continent

Over the years, there has been various suggestions on how African countries can better approach processes of product development, deployment, and commercialization of biotech products (Makinde et al., 2009; Glover et al., 2018; Akinbo et al., 2021). Most common in these suggestions, is the regulatory process by legislative means that needs to be agile, proactive towards advancing tools and mechanisms of biotechnology, and overall harmonisation of the various steps within the evaluation and decision-making processes. The development of biosafety legislation across African countries, has not seen much improvement or progress since 2016. However, the efforts of NEPAD in establishing the African Biosafety Network of Expertise (ABNE) Programme in 2009, has contributed immensely to assisting African countries to develop functional biosafety systems, followed by the implementation of the Cartagena Protocol on Biosafety. At regional level, both Economic Community of West African States (ECOWAS) and Common Market for Eastern and Southern Africa (COMESA) have made commendable efforts towards development and harmonization of biosafety regulations for their members (Akinbo et al., 2021). The envisaged action plans on biotechnology and biosafety are mainly geared towards increased investment and promoting economic trade opportunities in the region. The AUDA-NEPAD (African Union Development Agency–New Partnership for Africa Development), transformed in July 2018, has also initiated the establishment of the Integrated Vector Management (IVM) Programme to strengthen or build regulatory capacities to enable scientists to explore genetic engineering for potential novel vector control tools on the continent (Savadogo, 2022). According to NEPAD, one of the key IVM Programme objectives includes bringing together biosafety regulators and health-related regulators to ensure safe development and potential deployment of Genetically Based Vector Control innovative tools.

## Proposed coordinated approach for regions and the continent

The delay in the acceptance of GM crops in the African continent indicate that the introduction of similar or more advanced technologies, their envisaged benefits, their safety reservations/challenges and the associated safety guidelines should be addressed in a more transparent and coordinated manner to avoid a similar reaction towards NBT crops, that have already been adopted in some parts of the global north. So, policymakers should be given science-based information that would enable decision making in terms of biosafety, based on each country’s sovereign policies aiming at achieving the safe approval of GM crops and NBT/genome edited crops in the region, that would be environmentally and human safe and enable them to benefit from the advances in biotechnology (Akinbo et al., 2021). In the sections below, we identify areas where regions and the continent can work together, in a well-coordinated manner through a consultative approach towards advancing their biosafety regulations and biotechnology regulatory frameworks and policies.

### Identifying common needs and addressing them through dedicated networks

Across the four recognised African regions, the challenges and needs in terms of the economic advancement, addressing poverty, hunger, health and education are the same if not similar. The needs are in line with the African Union’s goals and priorities of Agenda 2063, whereby goal 3, 5 and 7, are specific to healthy and well-nourished citizens, modern agriculture for increased productivity and production, as well as environmentally sustainable and climate resilient economies and communities, respectfully (African Union Agenda, 2063, 2015). Furthermore, the Agenda 2063 links the various goals to the various Sustainable Development Goals (SDGs), an indication that the continent is geared towards realising a better and more sustainable future for all.

In this article, we have already demonstrated how biotechnology can help improve some of the current conditions for the African continent in the agriculture sector. Already, these regions address some of the political and economic challenges and conflicts they face through their joint regional committees, and the same should be done when it comes to other areas that are not necessarily political. Already, the AU-NEPAD Africa’s Science and Technology Consolidated Plan of Action (CPA) was adopted in 2005, reaffirming the continent’s collective action for using technological innovations (Makinde et al., 2009). The CPA work has been coordinated through the different centres, namely, 1) North African Biosciences Network (NABNet); 2) West African Biosciences Network (WABNet); 3) Southern African Network for Biosciences (SANBio) and 4) Biosciences eastern and central Africa Network (BecNet). Each of these centres (nodes) has its own focus area of work depending on the region’s needs aligned with various technological development and advancements. However, not much is known about these networks and what work they do or what their annual targets are in terms of their plans, focus work area and scope. Therefore, the goals of these networks need to be well communicated and coordinated across the regions so that those willing to get involved know how to do so. Also, there needs to be strong partnerships with various stakeholders and multidisciplinary teams to ensure efficiency and that all projects are implemented in a coherent manner.

### Being proactive through a horizon scanning initiative

Horizon scanning has been an effective tool to help adequately prepare for any future activities or for the anticipation of new challenges. If performed consistently, it can assist towards identifying the areas of needs, gaps, and there could be plans formulated towards addressing any of these. Also, horizon scanning is an effective tool for bringing different skills set and knowledge (expertise) in different subject areas together, to not only unpack common challenges, but to also find viable and sustainable solutions. Within the regions, initiatives such as the African Scientists Directory, administered by the Academy of Science of South Africa (Mark, 2020), can be used to bring different experts across the fields of biosafety and biotechnology together to work through any challenges or to plan ahead for Africa’s needs and challenges. Through such initiatives, capacity building can also be fast tracked by encouraging knowledge sharing and exchange of programs with the various institutions of higher education. However, it is important that participation in all of these forums and initiatives include all countries to make sure that no one is left behind.

### Addressing concerns on risks in the adoption and use of biotechnology

As already indicated, the African continent like many countries in the world is still grappling with the major areas of concern around the adoption and use of biotechnology. The major areas of concern remain, but not limited to the unintended harmful effects, environmental and food safety as well as ethical consideration. The social attitudes (and cultural aspects) also play a big role as they contribute to the public trust in the various processes governing the regulation and approval of GMOs on the continent. As a result, there remains strong doubts and to some degree prevalent acts of rebellion on any new form of biotechnology. Several studies have shown how the public is less aware and/or educated on the use and application of the technology across the continent (Zerbe, 2008; Clark et al., 2014; Gastrow et al., 2018). In some instances, it is also the general lack of understanding when it comes to the nature of genetic modification, its related techniques, and subsequent products (Marris, 2001; Aerni, 2013). It is also of note that even when such educational initiatives are put in place, there remains a greater degree of no interest, lack of participation or outright ignorance (Ahteensuu. 2012). Therefore, it remains an individual’s choice on how to receive and use the information at their disposal in the communication and debates related to the technology.

Other contributing factors relates to how the lack of transparency from governments is perceived by the public also contributes towards the erosion of trust on the newly deployed technologies. For example, the recent decision by the Kenyan government to lift a 10-year ban on GMOs brought about intense public opinion and debates (Oloo, 2022; The East African, 2022). Furthermore, it sparked *fears* that the country will be exposed to the control of seeds by multinational corporations, while biodiversity will continue to be at risk from GM crop cultivation. Also, the regulatory capacity was brought into question, with most activist groups and Non-Governmental Organizations (NGOs) believing that the country lacks the right approach to make the *correct decisions* on GMOs (Langat, 2022). Here, we witness once again the lack in proactiveness by regulatory authorities to take the public into their confidence in the decision taken on GMOs and addressing concerns on perceived risks. At the same time, we must acknowledge that it can also be difficult or close to impossible to try and convince the public to accept the decision on GMOs. However, it comes back to education and awareness, and the efforts to communicate transparently and in time, while allowing for a public participation process to take place. When such matters are debated vigorously in one country, it is bound to trickle to neighbouring countries and the region, making it difficult to manage any new ventures with the fear of the same (similar) setbacks. It is therefore important that the education and awareness on perceived risks associated with biotechnology be driven at regional level, with the help of experts in the field and the networks already established in the regions to deal with research and development of biotechnology.

### The need to prioritize

The African continent faces many challenges, yet the resources required to address many of the challenges are never adequate, especially in those countries that need them the most. This has over the years contributed to the growing gap between country advancements in many areas. While some countries continue to do well in the markets and other elements of trade and development, other countries continue to lag behind. Although the urgency to address certain challenges will vary from country to country, there are those that are common within the agriculture, environment and health sectors that affect countries similarly if not equally. Also, the impacts thereafter often means that countries end up assisting each other or relying on one another for certain services and/or aid. Therefore, through the use of tools such as the horizon scanning process, countries and regions can begin to narrow down on what needs to be done or achieved first, followed by a phased in plan and strategies of common interest and how to achieve them. The knowledge and expertise through the expert’s consultation would be critical for identifying the skills sets and resources needed to achieve the identified goals or priority areas. Central to this process, would be to identify the lead institutions or networks–per region, to champion the process. Here, various oversight, monitoring and reporting mechanisms would need to be in place for all reporting purposes and to account for any activities within the programs.

### Formulating a guided process on “process versus product” regulatory approach

The emerging and advancing biotechnology tools and methods have led to the regulatory authorities having to rethink the long-adopted approach of process-based regulations, previously developed for the GMO technology. In recent times, countries such as Argentina, Australia, Brazil, China, Japan, the United States, Nigeria, have taken the product-based approach (Lloyd et al., 2022). In both instances, the case-by-case basis evaluation in line with the CBD guidelines remains applicable. The debate is still out there in terms of the pros’ versus cons’ on the two regulatory approaches, but with the view that when it comes to CRISPR/Cas9-mediated (based) genome editing, there needs to be less regulatory burden as this hampers innovation; and this technology *only modifies* existing genetic material of the desired plant/animal (Lassoued et al., 2021). Therefore, the argument is that the same or similar regulations for GMOs, *should not* be subjected to genome edited products. For majority of the African countries (if not all), these new technologies are tried and licensed to foreign multinational companies and countries also remain importers of the “final product(s)”, derived through the new technologies.

As indicated, only seven (7) countries on the continent currently make use of the gene editing technology in various areas of research and development (Gakpo, 2021; Karembu, 2021; Sprink et al., 2022). Therefore, countries might remain net importers of GE derived products, making it difficult for them to apply the process-based risk analysis and regulations. Also, with the reality of the situation of porous borders between countries on the African continent where there is movement of people (including farmers), legally or illegally, may result in the exchange of seeds and food products where they are not approved or regulated formally. On the African continent, communities and small holder farmers have relied on informal seed systems for decades (Almekinders et al., 1994; Jones et al., 2001). This has served as a reliable and most important seed source of traditional food crops (Hlatshwayo et al., 2021). Furthermore, seed exchanges are central to the some of the traditional norms, are central to food sovereignty and strengthen social as well as cultural value systems among communities (van Niekerk & Wynberg, 2017). In addition, informal seed exchanges are not always restricted to or between farmers, as the practice can extend across villages or different regions (Pratap & Gupta, 2020).

Although the exchange of GM seeds or those developed using the technology is not established on the continent, it has been recorded that farmers do save GM derived seeds in South Africa (Masehela & Gouse, 2021). This makes it critical for countries to develop, finalise and implement their regulatory frameworks, and the process versus product regulatory approach will no doubt be central to deliberations involving the adoption and use of new technologies. As a result, countries and regions will need to engage in a more joint and coordinated manner to formulate their respective approaches in this regard, knowing very well that the option not to regulate, does not mean you will not have to deal with the product being present in the country.

### The political will, commitment, and action

While the field of biotechnology suffers from its own politics, the politics of governance–per country also needs to be decisive and favourable for research and development to thrive. It has been shown that government policies and positive political commitment to the biotechnology industry can have influence on how various investments are channelled for funding (Zarrilli, 2007). Africa also suffers from the formulation of many frameworks, action plans and the establishment of “working groups or committees”. Often, these groups come up with great regional approach and policy documents, which are signed off and endorsed by countries and regions, but hardly get implemented or reviewed for the effectiveness in terms of implementation. In some instances, no feedback is ever shared or given in terms of any progress or achievements. As a result, this adds to the frustrations in every attempt to fully implement biosafety regimes across the continent. Furthermore, managing public expectations becomes difficult as the overall public confidence and acceptance of biotechnology is pinned against the much-desired transparency and political goodwill.

Currently, there is a strong regional approach towards issues of trade (import/export) across the continent through the Inter Africa Trade discussions and policy developments, under the African Continental Free Trade Area (AfCFTA). These discussions also cover, to a large extent, country specific and regional orientated needs, challenges, and priorities. It is at this level that the biotechnology developments and advancements also need to take place, if they are to be taken seriously through any political agenda of the continent. Ultimately, harmonizing regulations and standards for biotechnology products, facilitating trade and economies is necessary for the advancement and adoption of new technologies in Africa.

## Concluding remarks

We are not the first authors to identify challenges in the acceptance and adoption of GMOs in the African continent. Also, pointing out that this currently impacts on how the new and emerging technologies are being view in the public domain. While the development and implementation of various biosafety regulations and policies remain a challenge for many African countries, a few have made good strides and have also started utilizing new technologies such as genome editing. This is because they realise the potential to harness the products that will benefit the countries towards addressing several challenges relating to, among others, economic growth and trade, the impacts associated with climate change, hunger and nutrition, crop diseases and pests, as well as health and pharmaceutical needs. All these developments cannot be successful if there is limited involvement of African scientists, regulators and policymakers in the development and harmonization of regulations and policies that favours the adoption and use of new and emerging technologies. It is for these reasons that we put forward a few consultative and collaborative based approaches that the countries, regions and continent must consider if they are to fully give the technology and its various developmental stages a chance on the African continent. Central to this proposal is the political will, commitment, and action. Ultimately, the scientists, regulators and policymakers need to come together and openly discuss how they view the impact of these technologies, address any reservations that potentially may cause delays in the implementation of regulatory frameworks and policies.

## Data Availability

The original contributions presented in the study are included in the article/supplementary material, further inquiries can be directed to the corresponding author.
